# Evaluation of targeted antimicrobial prophylaxis for transrectal ultrasound guided prostate biopsy: a prospective cohort trial

**DOI:** 10.1186/s12879-017-2470-1

**Published:** 2017-06-07

**Authors:** Teresa R. Zembower, Kelly M. Maxwell, Robert B. Nadler, John Cashy, Marc H. Scheetz, Chao Qi, Anthony J. Schaeffer

**Affiliations:** 1 Department of Medicine, Division of Infectious Diseases, Northwestern University Feinberg School of Medicine, 645 N. Michigan Ave., Suite 900, Chicago, IL 60611 USA; 2 Department of Urology, Northwestern University Feinberg School of Medicine, 303 E. Chicago Ave., Tarry 16-703, Chicago, IL 60611 USA; 3 Department of Pharmacy Practice, Midwestern University Chicago College of Pharmacy, 531 31st St., Alumni Hall 381, Downers Grove, IL 60515 USA; 4 Department of Pharmacy, Northwestern Memorial Hospital, 251 E. Huron St., Feinberg Pavilion LC 700, Chicago, IL 60611 USA; 5 Department of Pathology, Clinical Microbiology Division, Northwestern Memorial Hospital, 303 E. Chicago Ave., Ward 2-140, Chicago, IL 60611 USA

**Keywords:** Urology, Biopsy, Infection, Antibacterial agents

## Abstract

**Background:**

We evaluated the effectiveness of targeted antimicrobial prophylaxis in transrectal ultrasound guided prostate biopsy (TRUSP).

**Methods:**

A prospective, non-randomized cohort study was conducted. Rectal swab cultures plated on non-selective blood agar and on selective MacConkey agar supplemented with ciprofloxacin identified ciprofloxacin-susceptible and –resistant gram-negative bacteria (CS-GNB and CR-GNB). Patients with CS-GNB received ciprofloxacin while those with CR-GNB received directed prophylaxis. Infectious complications were defined clinically and microbiologically within 30 days after TRUSP. Data were derived at 7 and 30 days post procedure by questionnaires and electronic medical records. We hypothesized that there would be no difference in the infectious outcomes among the CS and CR groups.

**Results:**

From November 1, 2012 to March 31, 2015, 510 men completed the study; 430 (84.3%) had CS-GNB and 80 (15.7%) had CR-GNB. 484 (94.9%) completed the study per protocol, while 26 (5.1%) had an intention-to-treat (ITT) analysis. Of the 484, 475 (98.1%) had no infections, nine (1.9%) had infections, six of which (1.2%) were culture-proven (CP). The nine infections were as follows: five (1.0%) uncomplicated UTIs, one (0.2%) complicated UTI, and three (0.6%) urosepsis. One case of uncomplicated UTI and two cases of urosepsis were not CP, but were diagnosed clinically. ITT outcomes were similar. The infection rates were not statistically different between the CS-and CR-GNB patients (*p*-value = 0.314; 95% CI 0.8–3.3). The four patients with complicated UTIs or sepsis were hospitalized for a mean of 2.6 days and discharged without sequelae. Of the nine infections, three were antimicrobial prophylaxis failures (two ciprofloxacin and one amikacin); three were likely due to failure of the collection or processing of the rectal swab or increasing bacterial resistance between the time of swab collection and biopsy, and three developed clinical infections with no isolate recovered.

**Conclusions:**

Targeted antimicrobial prophylaxis follows the principles of antimicrobial stewardship and achieved a low rate of infectious complications with limited morbidity and no sequelae. This individualized method of prophylaxis may be widely applied. Further studies are needed to explore reasons for targeted prophylaxis failure and to determine comparative efficacy of non-ciprofloxacin-containing targeted prophylaxis regimens.

**Trial registration:**

ClinicalTrials.gov. NCT01659866. Registered 9 July 2012. First patient enrolled 1 November 2012.

**Electronic supplementary material:**

The online version of this article (doi:10.1186/s12879-017-2470-1) contains supplementary material, which is available to authorized users.

## Background

More than two million transrectal ultrasound guided prostate biopsies (TRUSP) are performed in the US and Europe annually [1, 2]. Infectious complications range from uncomplicated urinary tract infections (UTIs) to prostatitis to sepsis and death [3]. Empiric antimicrobial prophylaxis reduced the risk of infectious complications [4] but, with increasing resistance, infections after biopsy have risen [1, 5–9] and are most commonly fluoroquinolone (FQ)-resistant (R) gram-negative bacteria (GNB) [10].

Two primary prophylaxis strategies have emerged to prevent post-biopsy infections, a targeted or an empiric approach [7, 11–13]. The targeted approach involves obtaining a pre-biopsy rectal swab culture and choosing an antimicrobial agent based on culture results. The empiric approach is frequently based on local antibiograms [7, 12, 14–16]. Both approaches can be augmented by adding an antimicrobial agent to a fluoroquinolone or combination non-quinolone based regimen. To date, although studies have shown significant reduction in infectious complications, these studies may have been subject to underreporting because they were retrospective [5, 12, 14, 15], did not use phone contact for follow-up [7, 12–16], did not report non-hospitalized infections [7, 11, 12], and followed patients for less than 30 days [11, 16]. Additionally, the augmented approach introduces the concern for driving further antimicrobial resistance [17].

In our study, we prospectively evaluated patients by phone interview at 7 and 30 days to evaluate the rate of all post-biopsy infections in our patients treated with targeted prophylaxis based on rectal swab culture. Targeted antimicrobial prophylaxis achieved a low rate of infectious complications, limited morbidity and no sequelae in patients with either ciprofloxacin- susceptible or -resistant gram-negative rectal flora. These results suggest that this individualized method of prophylaxis may be widely applied.

## Methods

### Study setting and design

The study was conducted in the Northwestern University (NU) Feinberg School of Medicine Department of Urology at Northwestern Memorial Hospital between November 1, 2012 and March 31, 2015. This prospective, non-randomized cohort trial evaluated the efficacy of targeted pre-procedural antimicrobial prophylaxis for TRUSP and tested the hypothesis that infectious complication rates for targeted prophylaxis in patients with ciprofloxacin-susceptible compared to ciprofloxacin-resistant gram-negative bacteria (CS-GNB and CR-GNB, respectively) rectal flora would be equivalent. The Institutional Review Board of NU approved this study.

### Recruitment and eligibility criteria

Eligible patients were men 18 years or older selected to undergo TRUSP to evaluate for prostate cancer. Patients were excluded from the study if (1) they did not complete or withdrew informed consent; (2) their rectal swab cultures were CS-GNB but they did not receive ciprofloxacin as pre-procedure prophylaxis (i.e. ciprofloxacin-allergic patients); (3) their rectal swab cultures showed CR-GNB but they received ciprofloxacin; or (4) they did not complete the pre-biopsy questionnaire or the two post-biopsy phone screening evaluations. Of the 510 study participants, 26, who fulfilled eligibility criteria but received augmented prophylaxis (24 empiric, 2 directed) at the discretion of the treating physician, were evaluated in a separate intention-to-treat (ITT) analysis.

### Clinical specimen processing

After completing a pre-biopsy questionnaire (Additional file 1) to record demographics and to evaluate risk factors for infection, all subjects had rectal swab cultures obtained no more than 30 days prior to the TRUSP (Additional file 2). Swabs were cultured on blood agar and on MacConkey agar supplemented with 1 μg/ml ciprofloxacin (Thermo Scientific™ Remel™, Waltham, MA, USA). Quality control (QC) was performed on each new lot and shipment of MacConkey agar according to manufacturer’s instructions [18]. The following QC strains were utilized: *Staphylococcus aureus* ATCC® 25,923; *Pseudomonas aeruginosa* ATCC® 27,853; *Escherichia coli* ATCC® 25,922; and *Escherichia coli strain* #OC110. The plates were placed in a CO_2_ incubator (35° to 37 °C) and read at 36–48 h.

### Bacterial species identification and antimicrobial susceptibility testing

Blood agar plates without growth indicated inadequate specimen collections. Specimens with growth on blood agar only were considered to contain CS-GNR. Specimens with growth of gram-negative rods (GNRs) on blood and supplemented MacConkey agar underwent organism identification and antimicrobial susceptibility testing using an automated microbial system (Vitek® 2, Biomérieux, Durham, NC, USA) using the AST-GN47 cards. Antimicrobial susceptibilities were reported according to Clinical Laboratory Standards Institute (CLSI) guidelines [19].

### Selection of antimicrobial prophylaxis

Prophylactic antimicrobial agents were selected using a pre-determined protocol (Additional file 3). Patients with CS-GNR received ciprofloxacin 500 mg orally 2 h before TRUSP and 500 mg orally 12 h later. Subjects harboring CR-GNB received an antimicrobial agent based on the AUA guidelines (Additional file 3). Selection of the narrowest spectrum agent available was encouraged. Twenty-six patients (24 CS, 2 CR) received targeted prophylaxis augmented by another antimicrobial at the discretion of the treating physician based on the patient’s clinical status.

### Clinical evaluation

The data were derived from the pre-biopsy questionnaire and electronic medical record review. Charlson score was calculated by ICD-9 codes [20]. Immediately prior to the biopsy, enema and antimicrobial prophylaxis, including drug, dose, and timing, were confirmed. Phone screening at days 7 and 30 following TRUSP determined whether the patients experienced infectious complications (Additional file 4) or adverse drug reactions based on pre-determined criteria.

### Outcomes and definitions

The primary outcome was to compare the rate of infection following TRUSP in subjects with and without CR-GNB. Secondary objectives included determination of risk factors for infection and antimicrobial resistance traits of rectal swab isolates. Infectious complications were clinically defined as 1) uncomplicated urinary tract infection (UTI): dysuria, urgency, frequency or hematuria without fever and with or without pyuria (> 5 white blood cells per high-powered field or positive leukocyte esterase on urine dipstick) or bacteriuria (≥ 10^5^ colony-forming units/mL); 2) complicated UTI: fever, flank pain, nausea or vomiting with or without pyuria and bacteriuria; 3) urosepsis: criteria for sepsis, severe sepsis, and septic shock [21] were combined and categorized as urosepsis.

### Statistical analysis

Analyses were performed with SAS version 9.3 (SAS Institute Inc., Cary, NC) and R version 3.3 (R Foundation for Statistical Computing, Vienna, Austria). Categorical variables were described by percentages and compared using Chi-square or Fisher exact tests as appropriate. Continuous variables were described by means and evaluated using Student t-test. A *p*-value <0.05 was considered statistically significant. Our initial goal was to enroll 1700 patients, and have enough patients in each group to obtain a 95% confidence interval for the difference in the complication rates between the two groups with a margin of error of, at most, 2%.

## Results

### Clinical characteristics

**Fig. 1:**
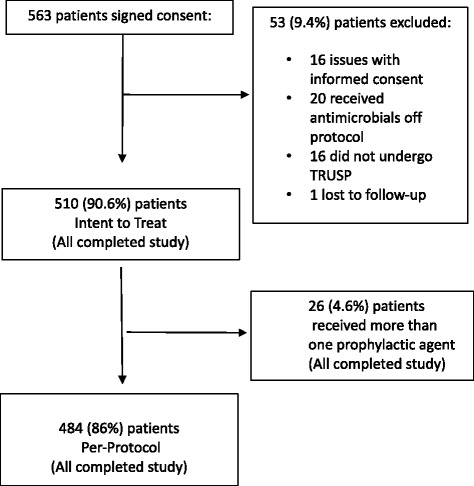
Study Subjects. TRUSP, Transrectal ultrasound guided prostate biopsy

Five hundred ten (90.6%) patients were included in the analysis (Fig. 1). 24 patients underwent more than one biopsy. Outcomes of all were recorded, but only infectious outcomes from the first biopsy were included. The time between rectal swab culture and TRUSP ranged from 7 to 30 days. Of the 510 patients, 26 received a second prophylactic antimicrobial agent at the discretion of the treating physician based on the patient’s clinical status; therefore, 510 patients were included in the ITT analysis and 484 in the per-protocol analysis.

Patient demographics and clinical characteristics of the 510 ITT patients stratified by ciprofloxacin susceptibility status are listed in Table 1. In this cohort, 430 (84.3%) had CS-GNB and 80 (15.7%) had CR-GNB on rectal swab culture. Univariate analysis demonstrated that patients with CS and CR rectal flora were similar except that a history of urinary tract infection was more common in CR patients (*p* = 0.004). Similar results were observed in the per-protocol population. (Data not shown).

**Table 1:** Demographics and clinical characteristics of the intent to treat study population stratified by ciprofloxacin susceptibility status

	Ciprofloxacin-susceptible *N* = 430 (84.3%)	Ciprofloxacin-resistant *N* = 80 (15.7%)	All *N* = 510	*P* value
Demographics				
Age, mean +/− SD, (range), years	62.7 ± 9.1 (33–88)	61.6 ± 7.6 (42–77)	62.5 ± 8.9 (33–88)	0.323
Race, n (%)				0.101
White	324 (75.3)	52 (65.0)	376 (73.7)	
Black	68 (15.8)	15 (18.8)	83 (16.3)	
Hispanic	21 (4.9)	9 (11.2)	30 (5.9)	
Other	17 (4.0)	4 (5.0)	21 (4.1)	
Clinical characteristics				
Reason for biopsy, n (%)				0.745
Elevated PSA	357 (83.0)	66 (82.5)	423 (82.9)	
Abnormal DRE	29 (6.7)	4 (5.0)	33 (6.5)	
Both	27 (6.3)	5 (6.2)	32 (6.3)	
Other	17 (4.0)	5 (6.2)	22 (4.3)	
Biopsy result, n (%)				0.848
Negative	219 (50.9)	38 (47.5)	257 (50.4)	
Prostate cancer	167 (38.8)	34 (42.5)	201 (39.4)	
HGPIN	44 (10.2)	8 (10.0)	52 (10.2)	
History of urinary tract infection, n (%)				0.004
Yes	39 (9.1)	16 (20.0)	55 (10.8)	
No	382 (88.8)	60 (75.0)	442 (86.7)	
Unknown/missing	9 (2.1)	4 (5.0)	13 (2.5)	
History of urinary retention; n (%)				0.769
Yes	33 (7.7)	6 (7.5)	39 (7.6)	
No	354 (82.3)	64 (80.0)	418 (82.0)	
Unknown/missing	43 (10.0)	10 (12.5)	53 (10.4)	
FQ usage in prior 2 years, n (%)				0.921
Yes	95 (22.1)	19 (23.8)	114 (22.3)	
No	225 (52.3)	42 (52.5)	267 (52.4)	
Unknown/missing	110 (25.6)	19 (23.8)	129 (25.3)	
Hospitalized in prior 1 year; n (%)				0.653
Yes	42 (9.8)	6 (7.5)	48 (9.4)	
No	374 (87.0)	73 (91.2)	447 (87.7)	
Unknown/missing	14 (3.3)	1 (1.2)	15 (2.9)	
Healthcare worker; n (%)				0.728
Yes	14 (3.3)	2 (2.5)	16 (3.1)	
No	357 (83.0)	70 (87.5)	427 (83.7)	
Unknown/missing	59 (13.7)	8 (10.0)	67 (13.1)	
Charlson comorbidity score; n (%)				0.693
0	348 (80.9)	68 (85.0)	416 (81.6)	
1	16 (3.7)	2 (2.5)	18 (3.5)	
2	32 (7.4)	3 (3.8)	35 (6.9)	
3–16	34 (7.9)	7 (8.8)	41 (8.0)	

*Abbreviations: PSA* prostate-specific antigen, *DRE* digital rectal exam, *HGPIN* high-grade prostatic intraepithelial neoplasia, *FQ* fluoroquinolone

### Microbiological characteristics

**Fig. 2:**
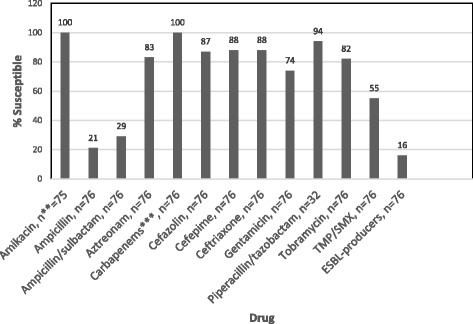
Antimicrobial Susceptibility Profile of 76* Ciprofloxacin-resistant E. coli Isolates. Tmp/smx, Trimethoprim/Sulfamethoxazole. *Not all isolates were tested for all antimicrobial agents, **Number of isolates tested for this particular antimicrobial agent, ***Carbapenems tested included imipenem (*n* = 49), meropenem (*n* = 76)

Of the 80 patients with CR-GNB, 76 (95%) harbored *Escherichia coli*, 2 (2.6%) *Pseudomonas aeruginosa*, 1 (1.2%) *Citrobacter freundii* and 1 (1.2%) *Klebsiella pneumoniae*. The antimicrobial susceptibility profiles of the CR-*E. coli* isolates are shown in Fig. 2. Using the Centers for Disease Control and Prevention (CDC) definitions [22] to classify the bacteria, 62 (79.5%) were multidrug-resistant (MDR), 17 (21.5%) were not MDR, and only 12 (15.2%) were resistant to ciprofloxacin alone. Of the MDR *E. coli*, 12 (15.2%) were extended-spectrum beta-lactamase producers (ESBLs). Interestingly, of the 24 patients who underwent two biopsies during the study period, the ciprofloxacin susceptibility status of 23 patients remained the same, while one patient who originally had CR-GNB had CS-GNB on subsequent culture. The antimicrobial prophylaxis regimens received are listed in Table 2. Oral regimens were used for 61 (76.3%) of the 80 patients harboring CR-GNB. Twenty-four patients with CS-GNB received prophylaxis with ciprofloxacin plus another empiric antimicrobial agent; likewise, 2 with CR-GNB received prophylaxis with two non-FQ agents directed by rectal flora susceptibility.

**Table 2:** Antimicrobial prophylaxis received versus ciprofloxacin resistance status

Drug received *N* = 510	Ciprofloxacin-susceptible 430, (84.3%)	Ciprofloxacin-resistant 80, (16.3%)
Amikacin	0	2 (2.5)
Aztreonam	0	1 (1.2)
Bactrim	0	23 (28.8)
Bactrim + Gentamicin	0	1(1.2)
Bactrim + Meropenem	0	1 (1.2)
Ceftriaxone	0	3 (3.8)
Cefuroxime	0	38 (47.5)
Ciprofloxacin	406 (94.4)	0
Ciprofloxacin + Amoxicillin	3 (0.7)	0
Ciprofloxacin + Gentamicin	21 (4.9)	0
Ertapenem	0	4 (5.0)
Gentamicin	0	7 (8.8)

### Infectious complications

The clinical characteristics stratified by infectious outcomes for the ITT and the per-protocol patients are shown in Tables 3 and 4, respectively. In the ITT group, 501 patients (98.2%) did not develop infections, while 9 (1.8%) had clinical infections and 6 (1.2%) of these were culture-proven (CP). In the per-protocol group, 475 patients (98.1%) did not develop infections, while 9 (1.9%) had clinical infections and again, 6 (1.2%) of these were CP. No statistically significant differences were observed in the demographics, clinical characteristics, or infection rate of the ITT versus the per-protocol analysis.

**Table 3:** Demographic and clinical characteristics stratified by infection outcomes in the intent to treat study population

	No infection *N* = 501 (98.2%)	Infection *N* = 9 (1.8%)	All *N* = 510	*P* value
Demographics				
Age, mean +/− SD, (range), years	62.5 ± 8.9 (33–88)	63.4 ± 6.5 (48–71)	62.5 ± 8.9 (33–88)	0.75
Race, n (%)				1.0
White	368 (73.5)	8 (88.9)	376 (73.7)	
Black	82 (16.4)	1 (11.1)	83 (16.3)	
Hispanic	30 (6.0)	0 (0.0)	30 (5.9)	
Other	21 (4.2)	0 (0.0)	21 (4.1)	
Clinical characteristics				
Biopsy result, n (%)				0.611
Negative	253 (50.5)	4 (44.4)	257 (50.4)	
Prostate cancer	196 (39.1)	5 (55.6)	201 (39.4)	
HGPIN	52 (10.4)	0 (0.0)	52 (10.2)	
History of urinary tract infection, n (%)				0.029
Yes	52 (10.4)	3 (33.3)	55 (10.8)	
No	437 (87.2)	5 (55.6)	442 (86.7)	
Unknown/missing	12 (2.4)	1 (11.1)	13 (2.5)	
History of urinary retention; n (%)				0.499
Yes	38 (7.6)	1 (11.1)	39 (7.6)	
No	410 (81.8)	8 (88.9)	418 (82.0)	
Unknown/missing	53 (10.6)	0 (0.0)	53 (10.4)	
FQ usage in prior 2 years, n (%)				0.678
Yes	111 (22.2)	3 (33.3)	114 (22.3)	
No	263 (52.5)	4 (44.4)	267 (52.4)	
Unknown/missing	127 (25.3)	2 (22.2)	129 (25.3)	
Hospitalized in prior 1 year; n (%)				0.400
Yes	46 (9.2)	2 (22.2)	48 (9.4)	
No	440 (87.8)	7 (77.8)	447 (87.7)	
Unknown/missing	15 (3.0)	0 (0.0)	15 (2.9)	
Healthcare worker; n (%)				0.713
Yes	16 (3.2)	0 (0.0)	16 (3.1)	
No	418 (83.4)	9 (100)	427 (83.7)	
Unknown/missing	67 (13.4)	0 (0.0)	67 (13.1)	
Charlson comorbidity score; n (%)				0.139
0	410 (81.8)	6 (66.7)	416 (81.6)	
1	17 (3.4)	1 (11.1)	18 (3.5)	
2	35 (7.0)	0 (0.0)	35 (6.9)	
3–16	39 (7.8)	2 (22.2)	41 (8.0)	

*Abbreviations: HGPIN* high-grade prostatic intraepithelial neoplasia, *FQ* fluoroquinolone

**Table 4:** Demographics and clinical characteristics stratified by infection outcomes in the per-protocol study population

	No infection *N* = 475 (98.1%)	Infection *N* = 9 (1.9%)	All *N* = 484	*P* value
Demographics				
Age, mean ± SD, (range), years	62.5 ± 9.0 (33–88)	63.4 ± 6.5 (48–71)	62.5 ± 9.0 (48–71)	0.75
Race, n (%)				1.0
White	351 (73.9)	8 (88.9)	359 (74.2)	
Black	77 (16.2)	1 (11.1)	78 (16.1)	
Hispanic	27 (5.7)	0 (0.0)	27 (5.6)	
Other	20 (4.2)	0 (0.0)	20 (4.1)	
Clinical characteristics				
Biopsy result, n (%)				0.609
Negative	241 (50.7)	4 (44.4)	245 (50.6)	
Prostate cancer	185 (38.9)	5 (55.6)	190 (39.3)	
HGPIN	49 (10.3)	0 (0.0)	49 (10.1)	
History of urinary tract infection, n (%)				0.029
Yes	50 (10.5)	3 (33.3)	53 (10.9)	
No	414 (87.2)	5 (55.6)	419 (86.6)	
Unknown/missing	11 (2.3)	1 (11.1)	12 (2.5)	
History of urinary retention; n (%)				0.511
Yes	38 (8.0)	1 (11.1)	39 (8.1)	
No	387 (81.5)	8 (88.9)	395 (81.6)	
Unknown/missing	50 (10.5)	0 (0.0)	50 (10.3)	
FQ usage in prior 2 years, n (%)				0.681
Yes	109 (22.9)	3 (33.3)	112 (23.1)	
No	254 (53.5)	4 (44.4)	258 (53.3)	
Unknown/Missing	112 (23.6)	2 (22.2)	114 (23.6)	
Hospitalized in prior 1 year; n (%)				0.388
Yes	44 (9.3)	2 (22.2)	46 (9.5)	
No	418 (88.0)	7 (77.8)	425 (87.8)	
Unknown/missing	13 (2.7)	0 (0.0)	13 (2.7)	
Healthcare worker; n (%)				0.710
Yes	16 (3.4)	0 (0.0)	16 (3.3)	
No	406 (85.5)	9 (100.0)	415 (85.7)	
Unknown/missing	53 (11.2)	0 (0.0)	53 (11.0)	
Charlson comorbidity score; n (%)				0.144
0	387 (81.5)	6 (66.7)	393 (81.2)	
1	16 (3.4)	1 (11.1)	17 (3.5)	
2	35 (7.4)	0 (0.0)	35 (7.2)	
3–16	37 (7.8)	2 (22.2)	39 (8.1)	

*Abbreviations: HGPIN* high-grade prostatic intraepithelial neoplasia, *FQ* fluoroquinolone

The infection outcomes stratified by CS or CR status for the ITT and per-protocol groups are shown in Table 5. All infections occurred in the per-protocol patients. There was no statistically significant difference in the rate of infections (CS = 1.5%; CR =3.9%; *p* = 0.337) between the two groups. Additionally, there were no statistically significant differences in the efficacy of the prophylactic regimens used. (Data not shown).

**Table 5:** Infectious outcomes of the intent to treat and per-protocol study populations stratified by ciprofloxacin susceptibility status

	Ciprofloxacin-susceptible	Ciprofloxacin-resistant	All	*P* value
Intent to Treat	*N* (%) = 430 (84.3)	*N* (%) = 80 (15.7)	*N* (%) = 510	
Any infection n, (%), 95% CI	6, (1.4), 0.5–3.8	3, (3.8), 0.8–10.6	9 (1.8) 0.8–3.3	0.314
No/Yes (n/n; %/%)	424/6 (98.6/1.4)	77/3 (96.2/3.8)	501/9 (98.2/1.8)	
Type of infection; n (%)				0.277
None	424 (98.6)	77 (96.2)	501 (98.2)	
Uncomplicated UTI	3 (0.7)	2 (2.5)	5 (1.0)	
Complicated UTI	1 (0.2)	0 (0.0)	1 (0.2)	
Urosepsis	2 (0.5)	1 (1.3)	3 (0.6)	
Per Protocol	*N* = 406 (83.9%)	*N* = 78 (16.1%)	*N* = 484	
Any infection n, (%), 95% CI	6, (1.5), 0.5–3.0	3, (3.9), 0.8–10.8)	9, (1.9), 0.9–3.5	0.337
No/Yes (n/n; %/%)	400/6 (98.5/1.5)	75/3 (96.1/3.9)	475/9 (98.1/1.9)	
Type of infection; n (%)				0.288
None	400 (98.5)	75 (96.1)	475 (98.1)	
Uncomplicated UTI	3 (0.7)	2 (2.6)	5 (1.0)	
Complicated UTI	1 (0.2)	0 (0.0)	1 (0.2)	
Urosepsis	2 (0.5)	1 (1.3)	3 (0.6)	

*Abbreviations: UTI* urinary tract infection

The characteristics of the 9 patients who developed infectious complications are shown in Table 6. All infections occurred in patients who received single drug targeted prophylaxis. Infectious complications occurred in 6 (66.7%) patients within 7 days and in 3 (33.3%) patients at 8, 11, and 13 days, respectively.

**Table 6:** Characteristics of patients with infections post-TRUSP

Type of infection	Age, Charlson score	Cipro	Abx prophy	Days to infxn	Positive culture? Culture site	Organism; susceptibilities	Treatment	Hospitalized? If so, LOS	Resolved	Comments
Uncomplicated UTI	65, 1	S	Cipro	1	Yes; Urine	*E. coli*: S cipro </= 1; R amp, pip, tcn, tmp/smx, gent, tobra	Ceftriaxone; cefuroxime	No; only seen in ED and discharged	Yes	Cipro failure
Uncomplicated UTI	64, 0	R	tmp/smx	2	Yes; Urine	E. coli: R tmp/smx	Ciprofloxacin	No	Yes	Failure of screening or increasing organism resistance
Uncomplicated UTI	62, 5	R	Amikacin	13	Yes; Urine	E. coli: S amikacin; R amp, amp/sul, cipro, tmp/smx, and ESBL	Unknown	No	Yes	Amikacin failure
Uncomplicated UTI	69, 0	S	Cipro	1	No	N/A	No antibiotics	No	Yes	Clinical diagnosis
Uncomplicated UTI	64, 0	S	Cipro	8	Yes; Urine	*K. pneumoniae*: R amp	Tmp/smx	No	Yes	Cipro failure
Complicated UTI	71, 4	S	Cipro	6	Yes; Urine	*P. mirabilis*: I cipro 2; R amp, nitro, tmp/smx:	Piperacillin; ceftriaxone; cefixime	No; 1 day of observation	Yes	Failure of screening or increasing organism resistance
Urosepsis	66, 0	S	Cipro	11	No	N/A	Ceftriaxone; cefuroxime	Yes; 2 days	Yes	Clinical diagnosis
Urosepsis	62, 0	R	tmp/smx	6	No	N/A	Piperacillin; meropenem; cefixime	Yes; 5 days	Yes	Clinical diagnosis
Urosepsis	48, 0	S	Cipro	1	Yes; Urine/blood	E. coli: ESBL plus I nitro, R cipro >4	Vancomycin and meropenem; Tmp/smx	Yes; 3 days	Yes	Failure of screening or increasing organism resistance

*Abbreviations: Abx prophy* antibiotic prophylaxis, *Amp* Ampicillin, *Amp/sul* Ampicillin-Sulbactam, *Cipro* Ciprofloxacin, *E. coli Escherichia coli*, *ED* emergency department, *ESBL* extended-spectrum beta-lactamases, *Gent* Gentamicin, *I* intermediate, *Infxn* infection, *K. pneumoniae Klebsiella pneumoniae*, *LOS* length of stay, *Nitro* Nitrofurantoin, *P. mirabilis* Proteus mirabilis, *Pip* Piperacillin, *R* resistant, *S* sensitive, *Tcn* Tetracycline, *Tmp/smx* Trimethoprim/Sulfamethoxazole, *Tobra* Tobramycin, *UTI* Urinary tract infection

The 5 patients with uncomplicated UTIs were managed as outpatients, 4 with and 1 without antimicrobial therapy; those with complicated UTIs or sepsis were managed with antimicrobial therapy as inpatients for 1–5 (mean 2.6) days.

All patients recovered without sequelae. There were no drug-related adverse events. None of the 26 patients in the ITT group who received augmented prophylaxis developed infectious complications.

Three (33.3%) of the 9 patients with infections were culture negative. Of the 6 patients with positive cultures, 3 (2 ciprofloxacin and 1 amikacin) were prophylaxis failures, i.e. the infecting bacteria were susceptible to the prophylactic drug they received. One patient who received ciprofloxacin was infected with bacteria with a MIC that was intermediate to ciprofloxacin and 2 patients, one who received trimethoprim/sulfamethoxazole and one who received ciprofloxacin, broke through with organisms fully resistant to these drugs.

## Discussion

With empiric single drug antimicrobial prophylaxis for TRUSP the hospitalization rate for infections is 0 to 6.3%.^2^ Two approaches to address this have been developed. Empiric augmented prophylaxis has shown initially promising results. However, it is often influenced by local hospital antibiograms, fails to assess the rectal source of the post-biopsy infections, and its use will likely be directly related to increasing antimicrobial resistance. Alternatively, rectal swab cultures can 1) determine the population of fluoroquinolone-resistant (FQR)-GNB in the rectal flora, 2) identify specific patients with FQ -GNB and 3) guide targeted single drug and augmented prophylaxis [7, 14, 16, 19, 23, 24]. The presence of FQ-R bacteria in the rectal flora constitutes a five-fold increase in the rate and potential severity of post-biopsy infections in patients receiving empiric fluoroquinolone prophylaxis [25]. This finding supports the utilization of pre-biopsy rectal cultures to identify patients at increased risk and to select targeted prophylaxis that is most likely to be effective. These principles were supported by this prospective cohort study, which showed a very low, i.e. 0.6%, sepsis rate and equivalent infectious complication rates among patients with CR-GNB or CS-GNB rectal flora who received targeted prophylaxis per our protocol.

In our study, the FQ resistance rate was 15.7%. We agree with Van Besien et al. [25] who stated that the benefit of targeted prophylaxis depends on local FQ-R prevalence rates. A randomized, blinded trial would subject the approximately 20% of patients who harbor FQ-R flora and receive FQ prophylaxis to the known 5-fold higher risk of infectious complications [25]. Similarly, empiric augmented prophylaxis could also subject patients to ineffective antimicrobial prophylaxis. For example, gentamicin is frequently used for augmented prophylaxis, but gentamicin resistance was present in 20% of the bacteria isolated from our patients with CR-GNB rectal flora (Fig. 2).

Our infectious complication rate in the per-protocol patients of 1.9% improved upon the 2.6% infection rate prior to the introduction of this protocol [14]. We used very stringent criteria: inclusion of all patients with symptoms of urinary tract infection irrespective of urine or blood cultures and phone screening at 7-and 30-days. Indeed, of the 9 patients identified, 3 had negative cultures before antimicrobial therapy was initiated and 3 were negative at 7 days but positive within 30 days. More importantly, only 4 (0.8%) had significant clinical infections and only 2 were culture-proven, febrile UTI (0.2%) and sepsis (0.2%).

Of the 9 patients with infections, only 3 had prior exposure to fluoroquinolones, a rate similar to those who did not become infected. Of the 6 patients with positive cultures, 3 were prophylaxis failures, i.e. the infecting bacteria was susceptible to the prophylactic drug given, (ciprofloxacin 2, and amikacin 1). Since all of these patients received their prophylaxis per protocol, we cannot implicate noncompliance of the patient or medical error. Breakthrough infections were probably due to excessive inoculum at the time of biopsy and/or an unrecognized host risk factor(s). Three patients were infected with bacteria with antimicrobial susceptibility breakpoints that showed resistance whereas the pre-biopsy rectal bacteria were susceptible. This could be due to sampling error of the rectal flora, lack of detection on the selective media or increasing antimicrobial resistance between the time of the culture and the biopsy. Although incorporating a lower concentration of ciprofloxacin into the screening media may have rendered the two rectal cultures with intermediate susceptibility ciprofloxacin-resistant, this extremely low incidence in our opinion would not support lowering the ciprofloxacin concentration in the screening media in our population.

Of the 6 culture-proven infections, the bacteria were multidrug resistant in 5, and of these, 2 were ESBLs. Williamson et al. noted similar results [26]. We identified trends in risk factors, e.g. history of UTI, however the incidence of infections was too low to achieve statistical significance [12, 24, 27–29].

This study is limited in that it was a single institution study and, for ethical reasons, was not blinded or controlled. Erectile dysfunction, a potential complication of TRUSP, was not evaluated, but it may occur as a result of inflammation induced by infection [30]. Thus, targeted prophylaxis could have other indirect benefits. Larger, multicenter studies are necessary to study this approach and its generalizability.

Additionally, this study does not claim superiority to empiric augmented prophylaxis in terms of infection reduction. However, it is likely that antimicrobial prophylaxis based on real time sensitivity data will be more durable and will likely outperform empiric prophylaxis as bacterial resistance inevitably increases. It is possible that more extended use of augmented prophylaxis or use of multiday therapy [16, 29, 31, 32] would have reduced our infectious complications further, but adherence to the guidelines of antimicrobial stewardship favors limited, single drug targeted prophylaxis for most patients.

## Conclusions

Targeted antimicrobial prophylaxis achieved a low rate of infectious complications in patients with CS- or CR- GNB rectal flora, limited morbidity and no sequelae. These results suggest that this individualized method of prophylaxis may be widely applied. Further studies are needed to explore reasons for targeted prophylaxis failure and to determine comparative efficacy of non-ciprofloxacin-containing targeted prophylaxis regimens.

## Additional files

Additional file 1: Pre-biopsy Questionnaire. This supplementary document details the data obtained from participants prior to biopsy to collect demographic information and to evaluate risk factors for infection. (DOCX 13 kb)

Additional file 2: Surveillance for Ciprofloxacin Resistant Enterobacteriaceae using MacConkey Agar with 1 μg/ml Ciprofloxacin. This supplementary document describes the protocol used for rectal swab culture in this study. (DOCX 16 kb)

Additional file 3: Antimicrobial Recommendations for TRUSP Prophylaxis. This supplementary document describes the protocol for selection of prophylactic antimicrobial agents. (DOCX 16 kb)

Additional file 4: Post-biopsy Phone Questionnaire. This supplementary document lists the questions asked of participants via phone following biopsy to assess for infectious complications and adverse drug reactions. (DOCX 71 kb)

## Abbreviations

Abx prophy: Antibiotic prophylaxis
Amp: Ampicillin
Amp/sul: Ampicillin-Sulbactam
AUA: American Urological Association
Cipro: Ciprofloxacin
CLSI: Clinical Laboratory Standards Institute
CP: Culture proven
CR-GNB: Ciprofloxacin-resistant gram-negative bacteria
CS-GNB: Ciprofloxacin-sensitive gram-negative bacteria
DRE: Digital rectal exam
*E. coli*: *Escherichia coli*
ED: Emergency department
ESBL: Extended-spectrum beta-lactamases
FQ: Fluoroquinolone
FQ-R: Fluoroquinolone-resistant
Gent: Gentamicin
GNR: Gram-negative rods
HGPIN: High-grade prostatic intraepithelial neoplasia
I: Intermediate
Infxn: Infection
ITT: Intent to treat
K. pneumonia: *Klebsiella pneumoniae*
LOS: Length of stay
MDR: Multi-drug resistant
Nitro: Nitrofurantoin
NU: Northwestern University
*P. mirabilis*: Proteus mirabilis
Pip: Piperacillin
PSA: Prostate-specific antigen
R: Resistant
S: Sensitive
Tcn: Tetracycline
Tmp/smx: Trimethoprim/Sulfamethoxazole
Tobra: Tobramycin
TRUSP: Transrectal ultrasound guided prostate biopsy
UTI: Urinary tract infection
