# Supplementary material for: Evaluation of targeted antimicrobial prophylaxis for transrectal ultrasound guided prostate biopsy: a prospective cohort trial
Source: BMC Infect Dis. 2017 Jun 7;17:401. doi: 10.1186/s12879-017-2470-1 (PMC5463462; doi:10.1186/s12879-017-2470-1)
Supplement: Supplementary file 1 — Pre-biopsy Questionnaire. This supplementary document details the data obtained from participants prior to biopsy to collect demographic information and to evaluate risk factors for infection. (DOCX 13 kb) [file 12879_2017_2470_MOESM1_ESM.docx]

**Additional file 1: Pre-biopsy Questionnaire**

**Subject # ___________**

**Case Report Form (CRF)**

**A: Pre-biopsy Questionnaire: Demographics, Co-morbidities, Infection Risk Factors:**

1. Age (in years) at study enrollment ________________________
2. Ethnicity [W, B, H, A, Other (specify)] ________________________
3. Reason for biopsy (elevated PSA, abnormal DRE, both, Other (specify): ______________________________________________________________________________
4. Charlson Score

_____________________

1. Have you taken a fluoroquinolone antibiotic Yes No Unknown (e.g., ciprofloxacin, levofloxacin, moxifloxacin) in the last 2 years?
2. If #5 is yes, when? If unknown, state this. ________________________
3. Have you taken another antibiotic in the last 2 years? Yes No Unknown
4. If #7 is yes, what drug(s) and when? If unknown, state this. ________________________________________________________________________
5. Have you been in a hospital or in a nursing home in the last year? Yes No Unknown
6. Are you a healthcare worker? Yes No
7. Have you had a prostate biopsy in the past? Yes No Unknown
8. If #11 is yes, when? If unknown, state this.___________________________________________
9. If #11 is yes, did you have an infection related to the prostate biopsy? Yes No Unknown
10. Do you have a history of urinary tract infection? Yes No Unknown

(This includes kidney, bladder or prostate infection)

1. Do you have a history of urinary retention? Yes No Unknown
