# Supplementary material for: Evaluation of targeted antimicrobial prophylaxis for transrectal ultrasound guided prostate biopsy: a prospective cohort trial
Source: BMC Infect Dis. 2017 Jun 7;17:401. doi: 10.1186/s12879-017-2470-1 (PMC5463462; doi:10.1186/s12879-017-2470-1)
Supplement: Supplementary file 2 — Surveillance for Ciprofloxacin Resistant Enterobacteriaceae using MacConkey Agar with 1 μg/ml Ciprofloxacin. This supplementary document describes the protocol used for rectal swab culture in this study. (DOCX 16 kb) [file 12879_2017_2470_MOESM2_ESM.docx]

**Additional file 2: Surveillance for Ciprofloxacin Resistant *Enterobacteriaceae* using MacConkey Agar with 1µg/ml** **Ciprofloxacin**

1. Purpose: To screen patients undergoing prostate biopsies for the presence of Ciprofloxacin resistant *Enterobacteriaceae*.
2. Specimen requirements and collection methods
   1. Specimen requirement
      1. A rectal swab is collected.
      2. To prevent contamination of the swab, it must be received in the laboratory closed and intact.
   2. Media requirement
      1. MacConkey agar with 1µg/ml Ciprofloxacin
         1. Store agar plates in a refrigerator at 2-8°C.
         2. Allow the temperature of agar plates to equilibrate before inoculation.
3. Materials and Reagents
   1. MacConkey Agar with 1µg/ml Ciprofloxacin - Remel # R110305
   2. Blood Agar (BAP)
   3. MacConkey Agar
   4. Oxidase reagent dropper
   5. Bacti-Cinerator
   6. 10µl calibrated inoculation loop
   7. O_2_ Incubator (35-37°C)
   8. Refrigerator (2-8°C)
   9. Tube rack
   10. Automated Identification System
   11. Vitek2 System
   12. *Staphylococcus aureus* ATCC^®^ 25923
   13. *Pseudomonas aeruginosa* ATCC^®^ 27853
   14. *Escherichia coli* ATCC^®^ 25922
   15. *Escherichia coli* strain #OC110
4. Inoculation and incubation of MacConkey Agar with 1µg/ml Ciprofloxacin
   1. Label the agar plate with a media label.
   2. Inoculate one third of the plate by rolling the swab.
   3. Streak the plate for isolation; flame the inoculating loop in the Bacti-Cinerator between each section.
   4. Incubate the inoculated plate in a 35-37°C, O_2_ incubator for 18 to 24 hours.
5. Temporary specimen storage
   1. Rack the swabs electronically in a tube rack after inoculation, and attach a rack label to the tube rack.
   2. Store the tube rack in the department’s specimen refrigerator at 2-8°C.
      1. Swabs are saved for seven days.
      2. Swabs are discarded in a biohazard receptacle after the seventh day.
6. Culture Examination
   1. Examine the MacConkey Agar with 1µg/ml Ciprofloxacin after 18 to 24 hours of incubation.
   2. If the culture is negative for growth, issue a preliminary report.
      1. Enter PRE in the Entry Field of Micro Result Entry in Cerner PathNet.
      2. A Preliminary Report dialog box will display. Enter the code **CIP** on the first line. It will display as follows:

*Culture in progress.*

- - 1. Click Verify.
    2. Incubate the agar plate for an additional 18 to 24 hours in a 35-37°C, O_2_ incubator.
  1. If there is growth on the MacConkey with 1µg/ml Ciprofloxacin, perform an oxidase on the colony. The oxidase should be negative for *Enterobacteriaceae*.
     1. Enter the quantity and colony description in the workup portion of Micro Result Entry.
     2. If the culture is mixed with different morphologies, subculture each morphology to MacConkey agar and incubate for 18 to 24 hours.
     3. Setup an organism identification on the automated identification system and an antibiotic susceptibility testing (AST) card on the Vitek2 system.
     4. Incubate the agar plate for an additional 18 to 24 hours in a 35-37°C, O_2_ incubator.
     5. Confirm that the organism is a member of the *Enterobacteriaceae* family. Non-*Enterobacteriaceae* organisms are reported as negative.
     6. Also, confirm that Ciprofloxacin (MIC ≥ 2) is intermediate or resistant. If Ciprofloxacin (MIC ≤ 1) is susceptible, the culture is reported as negative.
     7. Analyze the culture plate again after 36 to 48 hours of incubation.
     8. All culture plates are saved for seven days prior to disposal.

1. Reporting results
   1. Cultures negative for Ciprofloxacin resistant *Enterobacteriaceae*
      1. Cultures that are reported as negative include cultures negative for growth at 36 to 48 hours of incubation, cultures that grow non-*Enterobacteriaceae* organisms, and cultures that grow Ciprofloxacin susceptible (MIC ≤ 1) *Enterobacteriaceae* organisms.
         1. Enter “N” in the Entry Field of Micro Result Entry in Cerner PathNet.
         2. The negative result code will automatically populate the Final Report dialog box. It will display as follows:

*No ciprofloxacin resistant Gram negative bacilli isolated.*

- - - 1. Click Verify to final.
  1. Cultures positive for Ciprofloxacin resistant *Enterobacteriaceae*
     1. Enter FINAL in the Entry Field of Micro Result Entry.
     2. The Final Report dialog box will display. Enter the organism code in the first box on the first line. Enter **CIPR** in the second box on the first line, and **ISO** in the third box on the first line. This will add the phrase “*Ciprofloxacin Resistant isolated*.”
     3. Click Verify to final.

1. Quality Control
   1. Perform quality control on each new lot and shipment of MacConkey Agar with 1µg/ml Ciprofloxacin before they are used for patient testing.
   2. Plate the control organisms to Blood Agar plates from the frozen bullets in the -80°C freezer. Incubate in a 35-37°C, O_2_ incubator for 18 to 24 hours for growth.
      1. Use the following controls to perform QC on the MacConkey Agar 1µg/ml Ciprofloxacin.
         1. *Staphylococcus aureus* ATCC^®^ 25923 - No growth
         2. *Pseudomonas aeruginosa* ATCC^®^ 27853 - No growth
         3. *Escherichia coli* ATCC^®^ 25922 – No growth
         4. *Escherichia coli* strain #OC110 - Growth
      2. A sterility plate is set out at room temperature (19-25°C) and another sterility plate is set in a 35-37°C, O_2_ incubator.
      3. Document the QC results on the MacConkey Agar 1µg/ml Ciprofloxacin New Lot Quality Control Form (Attachment #1) after 18-24 hours of incubation. Document the receive date, manufacturer lot number, expiration date, and quantity received. Place a “√” under either A = acceptable or NA = not acceptable for sterility and performance. Document the date and technologist initials when quality control is complete.
      4. If the quality control does not yield the expected results, repeat the QC and document the corrective action. If the repeat testing fails, notify the Coordinator or designee immediately.
2. Procedure Limitations
   1. Proper collection techniques are required for accurate results.
3. Testing applies to Molecular Epidemiology technologists.
4. Attachments
   1. Attachment #1 *-* MacConkey Agar with1µg/ml Ciprofloxacin New Lot Quality Control Form
5. References
   1. Lautenbach, E., Harris, A., Perencevich, E., Nachamkin, I., Tolomeo, P., Metlay, J.

2005. Test Characteristics of Perirectal and Rectal Swab Compared to Stool Sample

for Detection of Fluoroquinolone-Resistant *Escherichia coli* in the Gastrointestinal

Tract. Antimicrobial Agents and Chemotherapy 49(2): 798-800.

1. Performance Standards for Antimicrobial Susceptibility Testing; Nineteenth

Informational Supplement Clinical and Laboratory Standards Institute.
