# Supplementary material for: Evaluation of targeted antimicrobial prophylaxis for transrectal ultrasound guided prostate biopsy: a prospective cohort trial
Source: BMC Infect Dis. 2017 Jun 7;17:401. doi: 10.1186/s12879-017-2470-1 (PMC5463462; doi:10.1186/s12879-017-2470-1)
Supplement: Supplementary file 3 — Antimicrobial Recommendations for TRUSP Prophylaxis. This supplementary document describes the protocol for selection of prophylactic antimicrobial agents. (DOCX 16 kb) [file 12879_2017_2470_MOESM3_ESM.docx]

**Additional file 3: Antimicrobial Recommendations for TRUSP Prophylaxis**

**Oral Options^a, b^**

| **Drug** | **Dose** | **Route/Schedule** | **Comment** |
| --- | --- | --- | --- |
| Trimethoprim-Sulfamethoxazole | 1 double strength tablet | Orally every 12 hours for 2 doses | ^a^Please note that while oral options may be available, they may not represent a therapeutic equivalent to ciprofloxacin which has a high oral bioavailability, a long drug half-life, and good tissue penetration into the prostate.  ^b^Please give oral regimens 2 hours prior to procedure and again 12 hours after the first dose. |
| Cefuroxime | 500 mg | Orally every 12 h for 2 doses |  |

*These options are interchangeable and selection is at the discretion of the treating physician based on

patient specific factors such as allergies and resistance patterns of the isolate.

**Intramuscular Options** ^a,b, c^

| **Drug** | **Dose** | **Route/Schedule** | **Comment** |
| --- | --- | --- | --- |
| Ceftriaxone | 500 mg dissolved in 1.8 mL of sterile water for injection, normal saline, or 1% lidocaine (withOUT epinephrine) | ONCE  intramuscularly | ^a^ Initiate Intramuscular treatment combination 2 hours prior to procedure.  ^b^ Use caution in patients with baseline nephrotoxicity or ototoxicity.  ^c^These options may not be preferable due to short drug half-lives (in patients with normal renal function); consideration should be given to providing an additional dose(s) post-procedure if 24 hours of antibiotic coverage is desired. |
| Aminoglycoside^b^ | Gentamicin 2 mg/kg  **or**  Amikacin 5 mg/kg given | ONCE  intramuscularly |  |
| Aztreonam^c^ | 500 mg dissolved in 1.5 mL (made by reconstituting the 1g vial with 3 mL of sterile water for injection or normal saline and delivering 1.5 mL) | Intramuscularly |  |
| Imipenem^c^ | 500 mg dissolved in 2 mL of 1% lidocaine (withOUT epinephrine) | Intramuscularly |  |
| Ertapenem | 1000 mg  Reconstitute the contents of a 1000 mg vial of ertapenem with 3.2 mL of 1.0% lidocaine HCL injection (withOUT) epinephrine | Intramuscularly |  |
| Meropenem | NOT AN FDA APPROVED  DOSING ROUTE |  |  |

*These options are interchangeable and selection is at the discretion of the treating physician based on patient specific factors such as allergies and resistance patterns of the isolate.

**Intravenous Options^a, b, c^**

| **Drug** | **Dose** | **Route/Schedule** | **Comment** |
| --- | --- | --- | --- |
| Ceftriaxone | 2000 mg | ONCE  Intravenously | ^a^Initiate 1 hour prior to procedure.  ^b^Use caution in patients with baseline nephrotoxicity or ototoxicity.  ^c^These options may not be preferable due to short drug half-lives (in patients with normal renal function); consideration should be given to providing an additional dose(s) post-procedure if 24 hours of antibiotic coverage is desired. |
| Aminoglycoside^b^ | Gentamicin 2 mg/kg  **or**  Amikacin 5 mg/kg given | ONCE  Intravenously |  |
| Aztreonam^c^ | 2000 mg | Intravenously |  |
| Imipenem^c^ | 1000 mg | Intravenously |  |
| Meropenem^c^ | 1000 mg | Intravenously |  |
| Ertapenem | 1000 mg | ONCE  Intravenously |  |

*These options are interchangeable and selection is at the discretion of the treating physician based on

patient specific factors such as allergies and resistance patterns of the isolate.
