# Supplementary material for: Evaluation of targeted antimicrobial prophylaxis for transrectal ultrasound guided prostate biopsy: a prospective cohort trial
Source: BMC Infect Dis. 2017 Jun 7;17:401. doi: 10.1186/s12879-017-2470-1 (PMC5463462; doi:10.1186/s12879-017-2470-1)
Supplement: Supplementary file 4 — Post-biopsy Phone Questionnaire. This supplementary document lists the questions asked of participants via phone following biopsy to assess for infectious complications and adverse drug reactions. (DOCX 71 kb) [file 12879_2017_2470_MOESM4_ESM.docx]

**Additional file 4: Post-biopsy Phone Questionnaire**

A: Pre-biopsy Questionnaire: Demographics, Co-morbidities, Infection Risk Factors:

1. Age (in years) at study enrollment

1. Ethnicity [W, B, H, A Other (specify)]
2. Reason for biopsy (elevated PSA, abnormal DRE, both, Other (specify):
3. Charlson Score:
4. Have you taken a fluoroquinolone antibiotic Yes No Unknown (e.g., ciprofloxacin, levofloxacin, moxifloxacin) in the last 2 years?
5. If #5 is yes, when? If unknown, state this.
6. Have you taken another antibiotic in the last 2 years? Yes No Unknown
7. If #7 is yes, what drug(s) and when? If unknown, state this.
8. Have you been in a hospital or in a nursing home in the last year? Yes No Unknown
9. Are you a healthcare worker? Yes No

11. Have you had a prostate biopsy in the past? Yes No Unknown

1. If #ll is yes, when? If unknown, state this. _
2. If #11 is yes, did you have an infection related to the prostate biopsy? Yes No Unknown
3. Do you have a history of urinary tract infection? (This includes kidney, bladder or prostate infection)

Yes No Unknown

1. Do you have a history of urinary retention? Yes No Unknown

Subject#____

**B. During the Biopsy:**

1. Did you take your enema? Yes No
2. Did you take your antibiotic prior to the biopsy? Yes No
3. If 17 is yes, which drug did you take?
4. If 17 is yes, when did you take it (military time)?
5. What time did the biopsy start (military time)?
6. **After the Biopsy:** Subject# ----

**C.1 Day 7 Outcomes Assessment –** Ask the following questions to determine if the patient

experienced signs and/or symptoms of infection following the biopsy; _

- 1. Did you have a fever (≥ 101°F) and/or an infection after the biopsy? Yes No

(An infection includes urinary tract/prostate infection, bloodstream infection), sepsis)

- 1. If the answer to #21 is No, **Stop Here.**
  2. If #21 is yes, when did fever/infection occur (preferably, record the date(s))? _________
  3. If you had a fever, did you take your temperature? Yes No
  4. If yes, what was your highest temperature?
  5. Did you experience shaking chills? Yes No

27. If yes, when (preferably, record the date(s))? ____________

1. Did you seek medical care for an infection? Yes No
2. If yes, where and when (record the institution name and date visited):
3. Were you diagnosed with an infection? Yes No
4. If yes, what type of infection (circle all that apply):
   1. Urinary tract infection
   2. Prostate infection
   3. Bloodstream infection
   4. Sepsis
   5. Other (specify):______________________________________________________________
5. Were you hospitalized for infection? Yes No
6. Do you have the name and contact information of the doctor who treated you? Yes No
7. Record the name and contact information of the treating physician.
8. May we contact your treating physician for more information/records? Yes No
9. If hospitalized, complete the SIRS criteria13 14 (circle all that applied on admission):

•

- 1. Temperature >100.4°F or less than 96.8°F
  2. Tachycardia > 90 beats per minute
  3. Respiratory rate >20perminute or a PaC02-<-32-mmHg
  4. White blood cell count > 12,000mm3 or <4,000 mm3 or>10% immature (band) forms

37. Cost of hospitalization: ___________________

1. How are you feeling now?

| a. | Fine, no problems |  |
| --- | --- | --- |
| b. | Still have signs/symptoms of an infection (Contact the treating Urologist if this is the  case) |  |
| c. | Other (specify): _____________________________________________________________ |  |

1. Did you have problems with the antibiotic that you took for the prostate biopsy? Yes No
2. If yes, specify according to **Appendix VI: Antimicrobial Recommendations for TRUSP Prophylaxis (reaction and grade):**

C.2 **Day 30 Outcomes Assessment** –Ask the following questions to determine if the patient experienced signs and/or symptoms of infection following the biopsy:

1. Did you have a fever (≥ 101°F) and/or an infection after the biopsy? Yes No (An infection includes urinary tract/prostate infection, bloodstream infection, sepsis)
2. If the answer to #41 is No, **Stop Here.**
3. If #41 is yes, when did fever/infection occur (preferably, record the date(s))? _
4. If you had a fever, did you take your temperature? Yes No
5. If yes, what was your highest temperature? _________________
6. Did you experience shaking chills? Yes No

. 47. If yes, when (preferably, record the date(s))? _

1. Did you seek medical care for an infection? Yes No
2. If yes, where and when (record the institution name and date visited):
3. Were you diagnosed with an infection? Yes No
4. If yes, what type of infection (circle all that apply):
   1. Urinary tract infection
   2. Prostate infection
   3. Bloodstream infection
   4. Sepsis
   5. Other (specify): _

52. Were you hospitalized for infection? Yes No

53. Do you have the name and contact information of the doctor who treated you? Yes No

54. Record the name and contact information of the treating physician.

__________________________________________________________________________________

55. May we contact your treating physician for more information/records? Yes No

56. If hospitalized, complete the SIRS criteria (circle all that applied on admission):

- 1. Temperature > 100.4°F or less than 96.8°F
  2. Tachycardia > 90 beats per minute
  3. Respiratory rate >20 per minute or a PaC02 < 32 mm Hg
  4. White blood cell count > 12,000mm3 or <4,000 mm3 or>10% immature (band) forms

1. Cost of hospitalization? ______________________
2. How are you feeling now?
   1. Fine, no problems
   2. Still have signs/symptoms of an infection (Contact the treating Urologist if this is the case)
   3. Other (specify): _
3. Did you have problems with the antibiotic that you took for the prostate biopsy? Yes No
4. If yes, specify according to **Appendix VI: Antimicrobial Recommendations for TRUSP Prophylaxis (reaction and grade):**

________________________________________________________________________

1. **Attachments:** Please de-identify and attach the following documents to the CRF.

L Rectal swab cx result

1. Was the isolate archived? Yes No

2. Biopsy report
